# Extra-Gastrointestinal Stromal Tumor of Prostate

**DOI:** 10.4274/balkanmedj.2015.1331

**Published:** 2017-03-28

**Authors:** Demet Etit, Haldun Kar, Neşe Ekinci, Ahmet Emre Yenipazar, Fulya Çakalağaoğlu

**Affiliations:** 1 Clinic of Pathology, Atatürk Training and Research Hospital, İzmir, Turkey; 2 Clinic of General Surgery, Atatürk Training and Research Hospital, İzmir, Turkey

**Keywords:** Extra gastrointestinal stromal tumor, Prostate, gist

## Abstract

**Background:**

Extra-gastrointestinal stromal tumor is defined as a mesenchymal neoplasm arising from soft tissues outside the gastrointestinal tract. Prostatic extra-gastrointestinal stromal tumor has rarely been noted.

**Case Report:**

A 56 year-old man presented with pain in the anal region. A digital rectal examination revealed that the prostate was markedly enlarged with a smooth, bulging surface. Computerized tomography images showed a 6 cm heterogeneous, infiltrative tumor within the prostate gland extending to the trigon of the bladder, left seminal vesicle and rectum. The tru-cut biopsy of the prostate was reported as leiomyoma. It was decided to perform surgery and the masses were easily and completely removed from the adjacent structures. The case was reported as extra-gastrointestinal stromal tumor within the intermediate- risk category with free surgical margins. Four years after the surgery, a locoregional failure was observed and treated with imatinib.

**Conclusion:**

Stromal tumor, although rare, should be considered in the differential diagnosis in patients with an enlarged prostate.

Gastrointestinal stromal tumor (GIST) is the most common mesenchymal malignancy of the digestive tract; approximately 70% originate from the stomach. It is thought that GISTs arise from the interstitial cells of Cajal. Extragastrointestinal stromal tumor (EGIST) is defined as a mesenchymal neoplasm arising from soft tissues outside the gastrointestinal tract, which is morphologically, histologically, and immunophenotypically similar to its gastrointestinal counterpart. Cajal-like cells have been described in the urinary tract and prostate, as well. Prostatic EGIST is rarely seen. It is defined as a mass in the prostate in radiologic imaging techniques. Diagnostic biopsy is essential in therapeutic approaches. The diagnosis of EGIST depends on the histopathologic features with immunohistochemical results. Indeed, immunohistochemistry has a major role in the differential diagnosis. Since their Cajal and/or Cajal-like cell origin, most of these tumors express KIT (CD117) tyrosine kinase and show the presence of activating mutations in KIT or platelet-derived growth factor receptor-α (1,2,3). Imatinib therapy alone or prostatectomy additional to imatinib therapy are the preferred methods for the treatment of prostatic EGIST. Here, we report a unique case of a primary EGIST of the prostate which was treated with enucleation without imatinib therapy and to our knowledge, resulted with the longest follow-up period.

## CASE PRESENTATION

**Figure 1:**
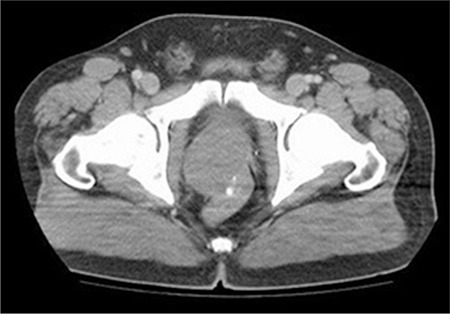
Computerized tomography: An enlarged prostate showing calcifications with expansive growth and compression of the rectum.

**Figure 2:**
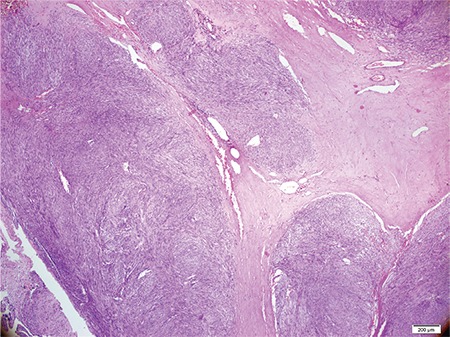
Spindle cell proliferation surrounding widened fibrous bundles enriched with vessels, HEX4.

**Figure 3:**
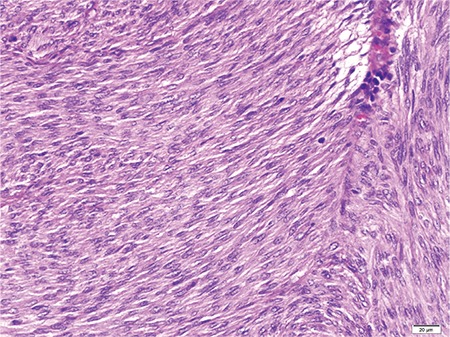
Tumor cells showing mild pleomorphism HEX20.

**Figure 4:**
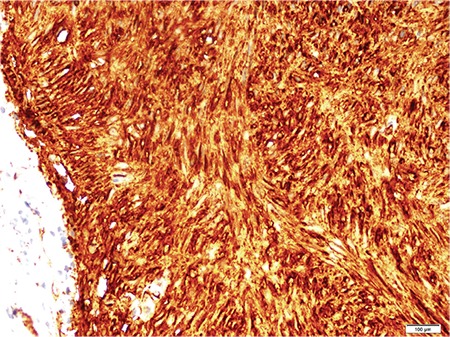
Immunohistochemical staining positivity for CD117 X10.

**Figure 5:**
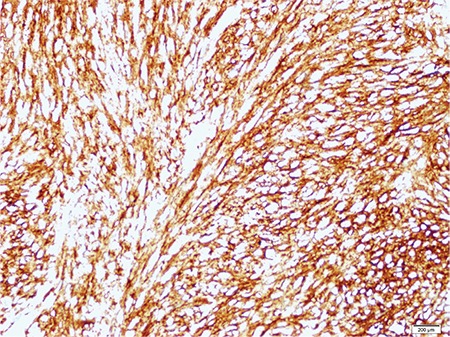
CD34 expression of the tumor cells X10.

In 2010, a 56 year-old man presented with pain in the anal region. His medical history was unremarkable. A digital rectal examination revealed a markedly enlarged prostate with a smooth, bulging surface. The serum prostate-specific antigen (PSA) level was 1.1 ng/mL. Computerized tomography (CT) images showed a 6 cm heterogeneous, infiltrative tumor within the prostate gland extending to the trigon of the bladder, left seminal vesicle and rectum (Figure 1). No lymph node involvement or any metastatic focus including bones was recorded. The colonoscopic examination revealed an extraluminal large asymmetric mass. The tru-cut biopsy of the prostate was reported as leiomyoma. At surgical observation, there were four seperate, circumscribed tumor masses located in the extraperitoneal, rectovesical and retroprostatic regions with no definitive association identified to the neighboring organs and structures. The masses were easily and completely removed from the adjacent structures. On macroscopic definition, the largest mass was measured 4 cm in diameter. The masses had well-defined borders with a pseudocapsule in shape and the cut surfaces were solid, firm and grayish-white. There were also some gritty areas in the macroscopic examination. Microscopic evaluation showed an infiltrative spindle cell proliferation with a mild cytologic atypia (Figure 2, 3, 4, 5). There were 4 mitosis per 50 high power fields (HPF) and there was no necrosis. The immunoprofile of the tumor was as follows: CD117 >50%: 3 (+), CD34 >50%: 1 (+), smooth muscle actin (SMA) <10% 1 (+), S-100 (-), desmin (-), Ki67 1% (Figure 5). The case was reported as EGIST within the intermediate-risk category with free surgical margins. A close follow-up was planned with CT scan for every three months in the first year and later for every 6 months. Because of the intermediate risk of the tumor, and the legislations regarding drug prescription rules in our contry, imatinib was not prescribed as a synchronous treatment. The patient was problem-free for the rest 49 months. While there was no significant finding in April 2014, eight months later, in November, the CT scan showed multiple nodules, with a greatest dimension of 3.5 cm - showing irregular contours with extraprostatic extension. Additionally, severe nodal involvement, including the left obturatory and right inguinal area, was reported. On positron emission tomography, both asymmetric masses in the right prostate lobe and lymph nodes showed high metabolic activity. Serum PSA level was 3.91 ng/mL. A fixed mass was palpated on the right lobe on digital rectal examination. The tru-cut biopsy of prostate had a spindle cell tumor consistent with EGIST. The immunostaining results were almost the same as the first specimen: SMA (+), CD117 (+) (1+) 100%, CD34 diffuse (+) 100%, smoothelin (-) some isolated cells, α-methylacyl coenzyme A racemase (-), Keratin AE1/AE3 mildly-focal (+), S100 (-), estrogen and progesterone receptor (-), desmin focal (+). Ki67 proliferation index was 1%. Mitotic figures were noted as 1/50 HPF. There was no necrosis. While two quadrants had 98% tumor, one had only 5% tumor. Because of the prostatic recurrence with nodal involvement, imatinib (Glivec; Novartis, Stein, Swiss) therapy was planned as a neoadjuvant therapy before radical surgery. A control CT showed regression in the tumoral masses in the 6^th^ month of follow-up and the patient has remained uneventful. Written informed consent has been obtained from the patient.

## DISCUSSION

GISTs almost exclusively occur in the gastrointestinal tract. The combination of c-kit and CD34 has become the most reliable marker for the diagnosis. EGIST constitutes 5-10% of GIST with a mean age of 58 years and the majority of locations emerge from the mesentery, omentum, and retroperitoneum (3,4,5,6,7). Noteworthy, various cases have also been validated in other sites, including the pancreas, urinary bladder, and seminal vesicles (8,9,10). In malignant prostatic tumors, sarcomas account for approximately 0.1% to 0.2%. Leiomyosarcomas and rhabdomyosarcomas are the most common types. Other primary sarcomas are extremely uncommon including EGIST, malignant fibrous histiocytoma, angiosarcoma, osteosarcoma, chondrosarcoma, malignant peripheral nerve sheath tumor, and synovial sarcoma. The first case defined as EGIST of the prostate was by Van Der Aa et al. (5). The age range varies from 31 to 75 in published reports. Symptoms were comprised of acute urinary retention, weight loss, abnormal rectal examination, perineal pain upon initiating the urinary stream, frequency, nocturia, dysuria, and hematuria (1,3,4,5,11,12). Our patient was a 56 year-old male with pain in the anal region.

The histomorphological features of EGIST are similar to the GIST. As known, GISTs are composed of spindle cells with prominent perinuclear vacuolization and may mimic smooth muscle tumors. Nuclear palisading in a myxoid stroma can be seen which may remind a nerve sheath tumor. Rarely, the tumor cells in GIST have epithelioid or pleomorphic type. Immunohistochemistry is very important in diagnosis. Strong and diffuse expression of CD117 is very limited in soft tissue sarcomas other than GISTs; 30-40% of cases are positive for CD34 and SMA, while very few are reactive for desmin and S-100 (13). The current tumor was positive for CD117 and CD34. Although prostatic stromal sarcoma seems a controversial entity, it should be kept in mind in the differential diagnosis. The main treatment for non-metastatic prostatic EGIST is radical prostatectomy. According to the English literature, patients undergoing radical surgery showed neither recurrence nor distant metastasis (Table 1). Enucleation surgery can be an alternative method which has a lower morbidity rate. Robb et al. (14) showed that enucleation was safe for esophageal GIST cases, especially when smaller than 65 mm, considering an intact pseudocapsule with negative microscopic margins preserved. This patient was free of disease for 49 months by enucleation with clear margins.

EGISTs are considered aggressive tumors in behavior with a high risk of recurrence. A proposal has been accepted for risk assessment in GISTs (Table 2) (15). In patients with totally excised primary EGISTs, determining risk factors such as size, cellularity and mitotic activity are important for postoperative assessment. The current patient was in the intermediate risk group with 1-4 mitosis per 50 HPF. Imatinib, a selective protein tyrosine kinase inhibitor, has been demonstrated to be an effective treatment for GISTs and EGISTs. Imatinib therapy is recommended for high risk patients following the complete surgical removal of EGIST.

## CONCLUSION

In summary, in patients with an “enlarged prostate”, EGIST should be considered in the differential diagnosis. Enucleation surgery with free margins can be performed in suitable tumors. Since there is a high recurrence risk, close follow-up is required. However, although imatinib can be the second choice after non-radical surgery in low/intermediate grade recurrent tumors, starting imatinib immediately after enucleation surgery should be considered in cases even for the low and intermediate risk groups for prostatic EGIST.

## Figures and Tables

**Table 1:**
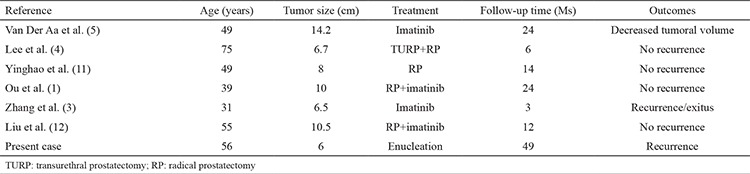
The treatment and outcomes of prostatic extra-gastrointestinal stromal tumors in the literature

**Table 2:**
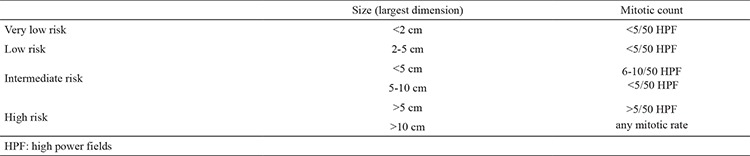
Risk of aggressive behavior in gastrointestinal stromal tumor
